# Does recovery reduce stigma? Icelanders’ attitudes toward individuals experiencing Schizophrenia and addiction

**DOI:** 10.1093/eurpub/ckaf260

**Published:** 2026-01-13

**Authors:** Sigrun Olafsdottir, Kari Kristinsson, Jon Gunnar Bernburg

**Affiliations:** Department of Sociology, University of Iceland, Reykjavik, Iceland; Department of Business, University of Iceland, Reykjavik, Iceland; Department of Sociology, University of Iceland, Reykjavik, Iceland

## Abstract

Public stigma toward individuals with mental illness and addiction remains a major barrier to treatment, recovery, and social integration. While previous studies have documented widespread negative attitudes, less is known about the role of recovery narratives in shaping stigma. This study draws on the 2025 Icelandic Stigma Study to examine whether descriptions of recovery reduce preferred social distance from individuals experiencing schizophrenia, alcohol addiction, or heroin addiction. Data were collected using a nationally representative online panel (*N = *1755). Respondents were randomly assigned to vignettes describing a character with one of the three conditions, with or without an added description of recovery. Preferred social distance was measured using a scale of eight items, and responses were analyzed with OLS regression models controlling for vignette characteristics and respondent demographics. Results show that descriptions of recovery significantly reduced preferred social distance across all conditions. The effect was strongest for alcohol addiction (33% reduction), followed by heroin addiction (23%) and schizophrenia (8%). Recovery narratives also reversed the relative ordering of conditions: while alcohol addiction was initially more stigmatized than schizophrenia, individuals in recovery from alcohol addiction were viewed more positively than those in recovery from schizophrenia. Female vignette characters elicited less social distance, while respondent characteristics had limited and inconsistent effects. The findings highlight the importance of recovery-oriented narratives in reducing stigma, particularly for addiction. Public campaigns that emphasize successful treatment and recovery may be especially effective in contexts such as Iceland, though condition-specific tailoring remains crucial.

## Background

In his seminal work, Goffman [1, p. 3] refers to stigma as “an attribute that is deeply discrediting.” To him, stigma can arise from physical deformities, blemishes of individual character and tribal stigmas. Ever since, scholars have attempted to understand how and why the public stigmatizes individuals experiencing mental health problems and addiction. Overall, less attention has been paid to stigma toward individuals experiencing drug addiction, as compared to mental illness, and existing data are often outdated [2]. Consequences of stigma have been documented for both types. They include resistance to seek treatment [3, 4], loss of self-esteem [5], social exclusion [6], struggles related to different spheres of live such as employment, housing and social relationships [7–9], and structural stigma manifested in less emphasis on funding for mental illness and addiction [10, 11].

Research in the USA has long illustrated stigmatizing attitudes toward individuals experiencing mental illness and addiction [12, 13] and this finding has been extended to a cross-national perspective. Specifically, the Stigma in Global Context-Mental Health Study (SGC-MHS) revealed widespread negative attitudes toward persons experiencing schizophrenia and depression across 16 countries on all continents of the world [14]. Similarly, research comparing Germany, Iceland and the USA showed considerable public stigma toward the same conditions, but less pronounced negative attitudes in Germany and Iceland than in the USA. [15]. This finding has been linked to the variation in media discourse across the three countries [16]. However, research has established that public attitudes toward persons with drug addiction are significantly more negative than toward those with mental illness in several countries, including the USA and Germany [13].

Societies have always responded in some ways toward what is constructed as “abnormal” [17]. The way mental illness and addiction is portrayed in society has implications for public views and how individuals experiencing these conditions are viewed and treated. Responses to mental illness have ranged throughout history from various forms of inhumane treatment [18] to mental illness being nothing but a myth [19] toward an increased emphasis on the biological foundations of mental illness and addiction [20–22] and increasingly to hope and recovery [23]. This more recent focus has been on the broader social context of the person experiencing the mental health problem, often with a focus on trauma. This has led to some agreement that mental illness and addiction are a complex mix of social and biological factors, for example that some people may be more vulnerable toward schizophrenia, but social factors impact whether this biological vulnerability manifests. Similarly, Pescosolido *et al.* [24] have shown that a specific genetic vulnerability to develop alcoholism is present only for males, those with weak social networks and those from economically disadvantaged backgrounds.

This variation in how mental illness and addiction is portrayed in society is mirrored in the focus of public campaigns to reduce stigma. A prominent approach, especially in the USA, during the last part of the 20th century was to promote the idea that mental illnesses are “a disease like any other” [25]. This aligned with the so-called genomic revolution, symbolized with the Human Genome Project that has been described as “the single most important project in biology and the biomedical sciences [26, p. 682].” Those who feared genetic essentialism raised concerns about genetic attribution increasing stigma, due to factors like the person being fundamentally different from others and that the problem being persistent and serious [27]. Research has shown that these concerns were at least partly accurate. For example, the period characterized by the “disease like any other” approach resulted in an increased acceptance of biological causes of mental illness among the public, yet no gains were made in reducing stigma among the US population [25, 28]. In addition, research indicates that it may even have increased stigma on certain domains, including the beliefs that the siblings and children of the person would develop the same disease [29].

These findings, in combination with other developments, shifted public campaigns toward an emphasis on personal narratives and recovery. A common approach was for prominent figures in society to “come out” and reveal their struggles with mental illness and/or addiction. These macro-level campaigns impact public views toward health problems. Experimental work has shown that portrayals of persons in recovery, whether for mental illness or for addiction, can meaningfully reduce stigma. Specifically, respondents exposed to vignettes describing individuals who transitioned to successful treatment reported significantly lower social distance than those exposed to untreated portrayals [30]. These findings support the notion that the public is responsive to narratives of treatability and recovery, even for conditions perceived as highly stigmatized, such as heroin addiction. This aligns with evidence from HIV-related stigma reduction, where public recognition of treatment effectiveness contributed to more supportive attitudes [2]. In essence, while beliefs on the causes underlying behavioral health problems may be neither relevant nor mutable in the public mind, the idea that individuals can be successful treated and recover may be. In fact, Goldman [31] went as far as to suggest that the abnormal behaviors portrayed in vignette studies represent a source of stigma, highlighting the importance of also evaluating levels of stigma associated with a character that undergoes a successful treatment for their condition.

Using data from the 2025 “Icelandic Stigma Study,” we evaluate the preferred social distance the public expresses from individuals experiencing schizophrenia and addiction to alcohol and heroin. Research has shown that levels of stigma are comparatively low in Iceland [14–16] and that approaches of recovery and empowerment have gained considerable support among the public and policy makers in Iceland [32]. With the current global focus on hope and recovery, we examine whether including recovery in our vignettes reduces social distance. Given research that shows that the public holds more negative attitudes toward those with addiction as compared to mental health problem [13] and that research has shown that the genetic revolution may made individuals with certain conditions less treatable in the publics’ mind, we expect the impact of recovery to vary depending on the condition presented. Finally, even though research has shown the diminishing role of social factors in determining levels of social distance since the last part of the 20th century [33], we do also evaluate whether social characteristic of the vignette character and the person evaluating the character impact social distance. In this paper, we ask three interrelated questions: (i) Does recovery from mental health problems or addiction reduce social distance from a person experiencing the condition; (ii) Does the impact of recovery vary by the condition presented; and (iii) Does social location of either the person in the vignette or the evaluator impact preferred social distance?

## Methods

We use the “2025 Icelandic stigma study” to evaluate the preferences Icelanders have for social distance and whether recovery has an impact. The survey builds upon the “Stigma in Global Context—A Mental Health Study” (SGC-MHS) that was collected in 18 countries in 2004–07. Iceland was one of the participation countries and a large proportion of the measures are adopted from the SGC-MHS (see e.g. [14]). The data were collected by Maskína, which is one of the main survey organizations in Iceland. The data were collected using the net-panel of Maskína which is designed to be a nationally representative sample of the Icelandic population. The number of respondents were 4866. We limit our analysis in this paper to respondents who received the schizophrenia, alcohol addiction and heroin addiction vignettes, with and without a recovery (*N = *1832). We deleted respondents with missing values on either the dependent or independent variables, resulting in an effective sample size of 1755. Studies of public attitudes do not require ethical approval in Iceland. The study adhered to ethical standards for research on human participants.

### Measures: the dependent variable

The dependent variable, preference for social distance from people with schizophrenia or addiction problems, is measured by responses to eight social distance items. Specifically, the respondents were asked to indicate how willing they would be to: (i) have a person described with a mental health problem as a neighbor; (ii) be friends with the person; (iii) spend time with the person; (iv) work closely with the person on a job; (v) hire the person for a job; (vi) have the person as a boss; (vii) have the person marry into the family; and (viii) have the person take care of your children or children that you know. Responses of “definitely willing,” “probably willing,” “don’t know,” “probably unwilling,” and “definitely unwilling” were coded 1 to 5, respectively, and combined to produce a summative scale of preferences for social distance from people with schizophrenia or addiction. The scale was divided by eight and ranges from 1 to 5. We only include respondents with a valid response on all eight items which reduces the number of respondents from 1832 to 1772. The internal consistency reliability coefficient (Cronbach’s alpha) for the 8-item scale was 0.933.

### Measures of independent variables: types of mental health problem, vignette characteristics, and characteristics of the evaluator

Tapping levels of prejudice toward people with mental health problems and toward the generic category of “mental illness” represents a methodological problem. For example, the public in many societies, including Iceland, has been sensitized to knowing what the “correct” responses toward mental illness are. We build on a strategy originally developed by the 1995 MacArthur Mental Health Module of the General Social Survey (see [12]) by using vignette strategy where schizophrenia, alcohol addiction, and addiction to heroin are described. To test the effect of recovery, we add a description of recovery at the end of each vignette for half of the sample. The vignettes are also varied by gender and immigration status. The schizophrenia vignette presented to respondents was the following, and we indicate both the gender and immigration variation and how recovery was added for some respondents.

Jón/Anna [Icelandic]/Ahmed/Rasha [Syrian] is an Icelandic/Syrian man/woman. Up until a year ago, life was pretty okay for Jón/Anna/

Ahmed/Rasha. But then, things started to change. He/She thought that people around him/her were making disapproving comments, and talking behind his/her back. Jón/Anna/Ahmed/Rasha was convinced that people were spying on him/her and that they could hear what he/she was thinking. Jón/Anna/Ahmed/Rasha lost his/her drive to participate in his/her usual work and family activities and retreated to his/her home, eventually spending most of his/her time on his/her own. Jón/Anna/Ahmed/Rasha became so preoccupied with what he/she was thinking that he/she skipped meals and stopped bathing regularly. At night, when everyone else was sleeping, he/she was walking back and forth at home. Jón/Anna/Ahmed/

Rasha was hearing voices even though no one else was around. These voices told him/her what to do and what to think. He/She has been living this way for 6 months.

*Recovery variation*: This situation lasted for 6 months but then he/she sought help. Since then Jón/Anna/Ahmed/Rasha has continued to seek help and is in recovery. The symptoms have been under control for the past year.

The two other vignettes are available in an online supplement. It is important to note that the vignettes focusing on addiction include additional description of recovery, specifically they state: “After completing treatment, he/she remained under the supervision of a counselor. After 3 months, he/she felt well enough to start working again. Since then (NAME) has continued to seek support and is in recovery.” Of course, we cannot exclude the possibility that this may impact responses. Each respondent received one version of the vignette and was then asked to answer about 60 questions about the person in the vignette.

We use four measures for respondent characteristics. Age is measured in years. Female is a binary variable (0 = male, 1 = female). One respondent identified as other and was deleted from the analysis. Marital status is measured with a binary variable (0 = not married, 1 = married or cohabiting). Education is measured by whether the respondent has obtained university education (0 = not a university degree, 1 = university degree).

### Analysis

We use descriptive statistics to show preferred social distance from vignette characters described with the three conditions. We then use an OLS-regression to evaluate significant differences between vignettes with and without recovery as well as to evaluate the impact of selected characteristics of the vignette character and the respondent on preferred social distance.

## Results

Table 1 tests our two first research questions, first whether adding a description of recovery significantly reduces preferred social distance. The answer is clearly yes, as including recovery is significant in model 1. Second, model 2 addresses whether the effect varies by condition. Again, our answer is yes. The impact of recovery on social distance is significantly more negative for both alcohol and heroin addiction than for schizophrenia. Thus, the preference for social distance decreases more for vignette characters described with addiction compared to schizophrenia. However, the tendency for recovery to reduce social distance does not differ between alcohol and heroin (*P* > .05).

**Table 1.** OLS-regression of preferred social distance on recovery and condition.^a^

	Model 1		Model 2	
	*β*	*P*	*β*	*P*
Recovery	−0.58	.00	−0.24	.00
Alcohol	0.09	.10	0.39	.00
Heroin	0.36	.00	0.58	.00
Alcohol × recovery			−0.59	.00
Heroin × recovery			−0.43	.00
Intercept	3.08		2.910	
Adjusted *R*^2^	0.10		0.12	

a The effect of recovery from alcohol vs. heroin is not significant (0.17, *P* = .14).

**Figure 1.**
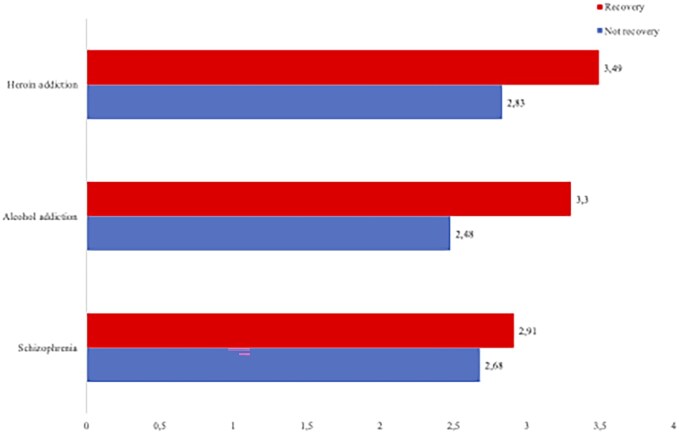
Preferred social distance from a character described with addictions and schizophrenia, with and without a description of recovery.

To illustrate, Fig. 1 shows the predicted values from model 2 (Table 1) for the social distance scale for the three conditions with and without the description of recovery. The figure shows that the public has less desired social distance toward individual with a description where recovery is included for all descriptions. For example, the mean for an individual described with heroin addiction is 3.49 but goes down to 2.82 if the description of recovery is added. The pattern holds for alcohol addiction and schizophrenia. Interestingly, recovery appears to have the strongest effect for the alcohol vignette. The Icelandic public prefers more social distance from a character described with alcohol addiction than schizophrenia. However, once recovery is included, the preferred social distance is less for a character with alcohol addiction in recovery than a character described with schizophrenia in recovery. Again, model 2 shows that the effect of recovery is significantly larger for alcohol and heroin than for schizophrenia, but the difference between heroin and alcohol is not significant. Specifically, including a description of recovery decreases the preferred social distance from schizophrenia by 8%, compared to 23% for heroin addiction and 33% for alcohol addiction.

To answer our third research question, Table 2 reaffirms that recovery reduces the social distance from all conditions, and it does so when the analysis controls for characteristics of the vignette character and selected characteristics of the respondent. The results across all models show that the public prefers less social distance from a female character in the vignette. Social locations of respondents does not appear to play a major role in explaining preferred social distance. As shown in model 1, women prefer less distance from a character described with schizophrenia. Our models can explain the greatest variation for the alcohol vignette in model 2 (*R*^2^ = .20) and there we find that older people prefer more social distance from a person experiencing alcohol addiction and those who have obtained a university degree prefer less social distance than those without a university degree. None of the characteristics of respondents are significant for the heroin vignette (model 3).

**Table 2.** OLS-regression of preferred social distance on vignette respondent characteristics.

	Model 1 Schizophrenia		Model 2 Alcohol		Model 3 Heroin	
	*β*	*P*	*β*	*P*	*β*	*P*
Vignette characteristics						
Recovery	−0.26	.00	−0.85	.00	−0.68	.00
Female	−0.26	.00	−0.19	.01	−0.18	.02
Immigrant	0.10	.18	0.01	.87	−0.03	.69
Respondent characteristics						
Age	−0.03	.28	0.05	.04	0.02	.37
Female	−0.18	.03	−0.11	.19	−0.05	.54
Married	−0.01	.94	−0.16	.08	0.02	.81
University	−0.06	.43	−0.21	.01	0.08	.33
Intercept	3.25		3.46		3.46	
Adjusted *R*^2^	0.04		0.19		0.10	

## Conclusion

This study set out to examine whether adding a transition to recovery influence Icelanders preferred social distance from individuals described as experiencing schizophrenia or addiction, and whether characteristics of either the vignette character or the respondent play a role in shaping these attitudes. Consistent with prior work emphasizing the importance of recovery in reducing to stigma [24], we find clear evidence that including a description of recovery significantly reduces the public’s preferred social distance across all conditions examined. This suggests that public perceptions are sensitive to cues about help-seeking and symptom management, highlighting the potential of recovery-oriented framings to reduce stigma.

Our findings show that this effect is particularly strong in the case of alcohol addiction, where recovery narratives not only lower stigma but reverse the relative ordering of the level of stigma attached to different conditions. While an individual described with schizophrenia is viewed more favorably than an individual described with alcohol addiction, the pattern reverses when recovery is introduced in the vignette. When recovery is introduced, an individual described with alcohol addiction is viewed more favorably than an individual described with schizophrenia. This pattern may reflect widespread familiarity with alcohol-related problems and a stronger belief in the effectiveness of treatment for alcohol misuse compared to other conditions. In contrast, while a recovery narrative reduces social distance for schizophrenia, the effect is smaller, suggesting that other factors—such as perceived dangerousness or unpredictability—may continue to shape public attitudes even in the presence of recovery cues.

As expected, we find limited evidence that social location of the respondent plays a consistent role in shaping preferred social distance. Some patterns emerge—for example, women are generally more accepting of individuals with schizophrenia, and university-educated respondents express less stigma toward alcohol addiction. However, these effects are modest and not uniform across conditions. This aligns with previous research showing that while demographic characteristics can shape attitudes, their explanatory power is often limited when compared to condition-specific beliefs and stereotypes [12, 24].

Interestingly, the gender of the vignette character was one of the few consistent predictors: female characters were met with less preferred social distance. This could reflect gendered perceptions of risk and morality, where women are seen as less threatening or more deserving of empathy. Such findings underscore the importance of considering how stigma intersects with gendered expectations and social norms.

Our study is not without limitations. First, previous work has relied on face-to-face data collection, once considered the state-of-the-art to collect high-quality social science survey data. We observe that respondents may be slightly more likely to skip answers using this method. However, this approach greatly decreases cost, making it feasible to collect survey data on stigma on regular basis in Iceland. Further, research has shown that respondents are more likely to disclose negative and stigmatizing attitudes using an online survey than the traditional interview [34]. Second, we use a net panel that is specifically designed to reflect the Icelandic population. It may have been more desirable to use the more standard method of recruiting our own sample based on the Icelandic census for the study. However, net panels are being increasingly used and have been found effective in evaluating public attitudes [35]. They are currently becoming the dominant approach in Iceland to collect social survey data across survey organizations. Third, there is a slight variation in the wording of recovery between the schizophrenia and addiction vignettes. We cannot rule out that this difference may have some impact on the association between recovery and social distance. However, it does not change our results that recovery significantly reduces stigma across all three conditions. Our research is the first study that we know of that empirically tests the impact of recovery. Future work should focus on disentangling what it is about recovery that reduces social distance across different conditions. Finally, the responses that are given to a vignette character may not completely reflect the actual responses of our respondents when confronted with an individual expressing similar symptoms in the community. However, as of now, vignette studies appear to be among the methods that are best suited to evaluate stigma among the general population [12, 14].

To conclude, our findings suggest that recovery-oriented narratives can be effective in reducing stigma, especially in the Icelandic context where the notion of recovery may resonate with broader values of self-sufficiency and rehabilitation. However, the variation across conditions and domains indicates that such narratives may work better for some groups than others. This may also indicate that the Icelandic public has a higher belief in recovery from addiction, especially alcohol addiction which may be due to high profile campaigns emphasizing the success of SÁA (Alcoholics anonymous in Iceland). A specific effort showing the potential of recovery from serious mental illness, such as schizophrenia is likely to be an effective strategy to reduce stigma [2, 29]. It is however crucial that public campaigns and clinical practices remain mindful of differences across conditions and groups, tailoring messages to address the specific beliefs and fears associated with each condition. Future research should explore how durable these recovery effects are over time and whether they translate into behavioral changes, such as greater social inclusion or reduced discrimination in institutional settings.

Conflict of interest: None declared.

## Supplementary Material

ckaf260_Supplementary_Data

## Data Availability

The data are not yet publicly available but are available from the corresponding author upon request. Key points Recovery narratives reduce the preferred social distance from individuals described with schizophrenia, alcohol addiction, and heroin addiction. The impact of recovery is stronger for addiction than for schizophrenia, reversing the relative stigma attached to these conditions. Female vignette characters elicit less social distance than male characters. Respondents’ social characteristics play only a minor role in shaping stigma. Public health campaigns that emphasize recovery may be particularly effective in reducing stigma and promoting social inclusion.
