# Supplementary material for: Does recovery reduce stigma? Icelanders’ attitudes toward individuals experiencing Schizophrenia and addiction
Source: Eur J Public Health. 2026 Jan 13;36(2):ckaf260. doi: 10.1093/eurpub/ckaf260 (PMC13016907; doi:10.1093/eurpub/ckaf260)
Supplement: ckaf260_Supplementary_Data [file ckaf260_supplementary_data.docx]

**Vignette descriptions for alcohol addiction and heroin addiction**

***Alcohol addiction***

(Jón/Anna/Ahmed/Rasha) is an (Icelandic/Syrian) (man/woman). More than a year ago, he/she began drinking more alcohol than he/she was used to. He/she also noticed that he/she needed to drink twice as much as before to get the same effect. He/she has tried several times to cut down or stop drinking, but was unable to. Each time he/she tried to cut back, he/she became very restless, sweaty, and had trouble sleeping, so he/she had another drink. The family complained that he/she was often hungover and unreliable – making plans one day and canceling them the next. This situation lasted for six months.

***With recovery***

He/she went into treatment and addressed the problem. After completing treatment, he/she remained under the supervision of a counselor. After three months, he/she felt well enough to start working again. Since then, (Jón/Anna/Ahmed/Rasha) has continued to seek support and is in recovery. His/her symptoms have been under control for the past year.

***Heroin addiction***

(Jón/Anna/Ahmed/Rasha) is an (Icelandic/Syrian) (man/woman). More than a year ago, (NAME) went to a party and used heroin for the first time. After that, he/she began to use heroin regularly. At first, he/she only used it at parties on weekends, but after a few weeks he/she felt an increasing need for more. (NAME) began using heroin two or three times a week. He/she spent all his/her savings and borrowed money from friends and family to buy more heroin. Each time (NAME) tried to reduce use, he/she became anxious, sweaty, nauseous for many hours, and could not sleep. These symptoms continued until he/she used heroin again. (NAME)’s friends complained that he/she was unreliable – making plans one day and canceling them the next. His/her family said that he/she had changed and that they could no longer rely on him/her. This situation lasted for six months.

***With recovery***

He/she went into treatment and addressed the problem. After completing treatment, he/she remained under the supervision of a counselor. After three months, he/she felt well enough to start working again. Since then, (NAME) has continued to seek support and is in recovery. His/her symptoms have been under control for the past year.
